# Prediction of Age-Adjusted Mortality From Stroke in Japanese Prefectures: Ecological Study Using Search Engine Queries

**DOI:** 10.2196/27805

**Published:** 2022-01-20

**Authors:** Kazuya Taira, Sumio Fujita

**Affiliations:** 1 Department of Human Health Sciences Graduate School of Medicine Kyoto University Kyoto Japan; 2 Yahoo Japan Corporation Tokyo Japan

**Keywords:** stroke, age-adjusted mortality, search engine query, Japan, random forest, generalized linear mixed model, search engine, GLMM, information-seeking behavior

## Abstract

**Background:**

Stroke is a major cause of death and the need for nursing care in Japan, with large regional disparities.

**Objective:**

The purpose of this study was to clarify the association between stroke-related information retrieval behavior and age-adjusted mortality in each prefecture in Japan.

**Methods:**

Age-adjusted mortality from stroke and aging rates were obtained from publicly available Japanese government statistics. A total of 9476 abstracts of Japanese articles related to symptoms and signs of stroke were identified in Ichushi-Web, a Japanese web-based database of biomedical articles, and 100 highly frequent words (hereafter referred to as the Stroke 100) were extracted. Using data from 2014 to 2019, a random forest analysis was carried out using the age-adjusted mortality from stroke in 47 prefectures as the outcome variable and the standardized retrieval numbers of the Stroke 100 words in the log data of Yahoo! JAPAN Search as predictive variables. Regression analysis was performed using a generalized linear mixed model (GLMM) with the number of standardized searches for Stroke 100 words with high importance scores in the random forest model as the predictive variable. In the GLMM, the aging rate and data year were used as control variables, and the random slope of data year and random intercept were calculated by prefecture.

**Results:**

The mean age-adjusted mortality from stroke was 28.07 (SD 4.55) deaths per 100,000 for all prefectures in all data years. The accuracy score of the random forest analysis was 89.94%, the average error was 2.79 degrees, and the mean squared error was 13.57 degrees. The following 9 variables with high importance scores in the random forest analysis were selected as predictive variables for the regression analysis: male, age, hospitalization, enforcement, progress, stroke, abnormal, use, and change. As a result of the regression analysis with GLMM, the standardized partial regression coefficients (β) and 95% confidence intervals showed that the following internet search terms were significantly associated with age-adjusted mortality from stroke: male (β=−5.83, 95% CI −8.67 to −3.29), age (β=−5.83, 95% CI −8.67 to −3.29), hospitalization (β=−5.83, 95% CI −8.67 to −3.29), and abnormal (β=3.83, 95% CI 1.14 to 6.56).

**Conclusions:**

Stroke-related search behavior was associated with age-adjusted mortality from stroke in each prefecture in Japan. Query terms that were strongly associated with age-adjusted mortality rates of stroke suggested the possibility that individual characteristics, such as sex and age, have an impact on stroke-associated mortality and that it is important to receive medical care early after stroke onset. Further studies on the criteria and timing of alerting are needed by monitoring information-seeking behavior to identify queries that are strongly associated with stroke mortality.

## Introduction

### Research Background

In Japan, approximately 106,000 people die from stroke per year, making it one of the leading causes of death [1]. Furthermore, stroke, a cerebrovascular disease, is at 15.1%, the second leading reason for the need for nursing care, following dementia, at 18.7% [2]; additionally, some of these patients develop dementia, an organic sequela of stroke. The increase in the number of people in need of nursing care not only affects patients’ quality of life, but also causes problems, such as the burden of nursing care on caregivers [3]. In addition, Japan's social and economic losses, such as increased social security costs [4] and productivity losses due to the inability of caregivers to work in the labor market [5], are significant. Thus, stroke and dementia prevention are important for the prevention of conditions requiring nursing care.

Due to advances in treatment methods, such as the administration of tissue plasminogen activator and thrombus recovery therapy, stroke has become a disease with an improved prognosis and reduced sequelae if treated promptly after onset [6]. However, regional differences in the outcome of stroke treatment have been reported [7], and it is important to understand the causes of such differences to reduce health disparities and extend healthy life expectancy.

### Prior Work

The area of research known as infodemiology, which uses user-contributed health information to improve public health, has attracted attention in recent years [8,9]. Several ecological studies have reported an association between the behavior of seeking stroke-related information on the internet and stroke incidence or mortality. Walcott et al [10] reported that in the United States, the web search interest level of common queries used to identify signs and symptoms of stroke and to educate people about stroke correlates with the relative stroke prevalence. Kumar et al [11] described a correlation between the relative search volume of queries related to cardiovascular disease and the burden of cerebrovascular disease mortality in the United States and Australia [11]. Furthermore, an association between the normalized search volume for stroke and stroke-related queries and the incidence of stroke has been reported in Malaysia [12]. These studies also suggest that internet search behavior is associated with the tendency for stroke to be more common in winter because of the nature of the disease [11,12].

In addition to stroke, other major causes of death, such as cancer and heart disease, have also been reported to be associated with internet search behavior. Hospitalization rates for coronary heart disease are associated with the number of search requests for symptoms experienced by these patients, especially chest pain [13], and cancer incidence and mortality are associated with the search volume of cancer types normalized to the total Google search volume [14].

### Motivation for This Study

Stroke-focused ecological studies have been conducted in the United States, Australia, and Malaysia [10-12], but have not been reported in Japan before. In Japan, stroke is a major cause of death and the need for long-term care; thus, equivalent studies are needed. In addition, clarification of the relationship between information retrieval behavior and stroke may enable real-time regional monitoring, leading to early detection and treatment of patients affected by stroke and the resolution of regional disparities [7].

### Aim of This Study

The purpose of this study was to clarify the association between stroke-related query retrieval behavior and age-adjusted mortality in each prefecture in Japan and to assess the impact of each query on age-adjusted mortality from stroke using regression analysis. Based on these findings, this study provides suggestions to improve decision-making processes in public health.

## Methods

### Search Queries Related to Stroke Signs and Symptoms

To identify search queries most likely to be related to stroke, 100 frequently occurring words were extracted from the abstract of Japanese academic papers related to stroke. The abstracts were retrieved using Ichushi-Web [15], a fee-based, web-based database of medical literature in Japan. A total of 9476 abstracts from original articles containing the keywords “stroke” and “signs and symptoms” were included (Table 1). Since we considered that words with statistically significant associations to stroke may have a higher predictive accuracy, to improve their extraction, unnecessary information, such as the citations list and the conflicts of interest declaration, was not included. Furthermore, the abstract of the academic paper, which represents the authors’ key messages, was judged to be sufficient.

Morphological analysis was performed on these 9476 abstracts and 100 frequently occurring words (hereafter referred to as the Stroke 100 words) were identified (Multimedia Appendix 1). In the morphological analysis, an ipadic-NEologd dictionary (mecab-ipadic-NEologd) was used as a dictionary, and the words included in the stop word list provided by Apache Subversion (Apache Software Foundation) [16], as well as the words “abstract”, “introduction”, “method”, “result”, and “conclusions” (depending on the data to be analyzed), were excluded.

**Table 1:** Retrieval strategy for abstracts in Ichushi-Web and search results.

Strategy number	Retrieval strategy	Number of search results
1	Stroke^a^	134,111
2	Signs and symptoms^a^	1,162,123
3	1 and 2	36,640
4	Original articles in 3 whose abstracts could be downloaded	9476

^a^Abstracts containing these keywords were retrieved. Synonyms of keywords found in a thesaurus search were also included in the retrieval strategy. No field restrictions were used.

### Outcome Variables

Age-adjusted mortality from stroke by prefecture in Japan was used as the outcome variable. Age-adjusted mortality rates were calculated using the direct method based on the number of stroke deaths extracted from vital statistics provided by the Japanese government [1], and the total population was derived from population estimates or the national census. The reference population used for age adjustment was the 1985 model population, in accordance with Japanese practice [17]. These data can be obtained from e-Stat [18], a portal site that compiles Japanese government statistics. For variables used in the analysis, data from all 47 prefectures were combined vertically for the 6 years from 2014 to 2019 and analyzed as a variable with a sample size of 282 (47 prefectures×6 years).

### Predictive Variables

For the identified Stroke 100 words, we extracted the number of search requests in the 47 prefectures from 2014 to 2019 using search log data from Yahoo! JAPAN Search, one of the major search engines in Japan. Since the actual number is strongly affected by words with consistently high search volumes and the population size of the prefecture, the number of searches were converted to a standardized characteristic score as a predictive variable based on equation (2). UU is the number of unique users.



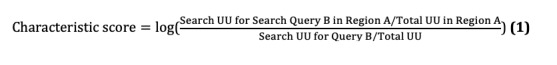





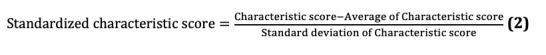



### Control Variables

As control variables, the data year, prefectures, and the aging rate for each prefecture were used. The data year variable included the 6 years from 2014 to 2019, with values converted from 1 to 6 in ascending order. The prefecture variable included all 47 prefectures in Japan and was converted to a numerical value where each prefecture from north to south was assigned a number from 1 to 47. The aging rate for each prefecture was calculated using the population census or population estimate statistics, and the ratio of the population aged 65 years or older was used.

### Statistical Analysis

A random forest regressor analysis was performed using age-adjusted mortality from stroke as the outcome variable and the Stroke 100 words as predictors. The data were divided into 70% training data and 30% test data; the modeling was conducted with the training data, and the evaluation of the model was conducted with the test data. In the modeling, 3-fold cross-validation was performed, and the hyperparameter was tuned using grid search. The hyperparameter settings used in the grid search are shown in Textbox 1. For the model with the best parameters, the accuracy score was calculated. In addition, high-importance variables were identified using the Gini importance score, and predictive variables for regression analysis were selected.

Regression analysis using a generalized linear mixed model (GLMM) was performed because it was not possible to determine which queries were associated with age-adjusted mortality from stroke in prefectures in the random forest regression. First, the Pearson correlation coefficient and variance inflation factor (VIF) were calculated for age-adjusted mortality from stroke, prefectural variables, data years, aging rates, and queries as predictors selected in the random forest analyses, and it was confirmed that no problems arose from multicollinearity. In the GLMM, age-adjusted mortality due to stroke was used as the outcome variable, queries selected in the random forest analysis were used as predictors, the aging rate and data year were used as control variables, and the random slope of data year and random intercept were calculated by prefecture.

To estimate the parameters, simulated draws from the posterior were obtained for each parameter using the Markov chain Monte Carlo (MCMC) method [19,20]. Then, Rhat was calculated to confirm the convergence of the simulation. Rhat is an index of divergence among chains; in the case of 3 or more chains, a value of 1.1 or less is by convention considered to indicate convergence.

Analyses were performed using the scikit-learn Python library (Python Software Foundation) and the open-source statistical software R, version 3.6.2 (R Foundation for Statistical Computing). The Rstan package was used for parameter estimation by MCMC.

**Hyperparameter values used to tune the random forest regression by grid search.**

- Number of trees in the forest (n_estimators): 10, 20, 30, 40, 50, 60, 70, 80, 90, 100, 110, 120, 130, 140, 150, 160, 170, 180, 190, 200
- Bootstrap: True, False
- Function to measure the quality of the split (criterion): mean squared error (MSE), mean absolute error (MAE)
- Maximum depth of the tree (max_depth): 10, 20, 30, 40, 50, 60, 70, 80, 90, 100, None
- Number of features to consider when looking for the best split (max_features): Auto, Square root, log2
- Minimum number of samples required to be at a leaf node (min_samples_leaf): 1, 2, 4
- Minimum number of samples required to split an internal node (min_samples_split): 2, 5, 10

## Results

### Characteristics of Government Statistics from e-Stat

**Figure 1:**
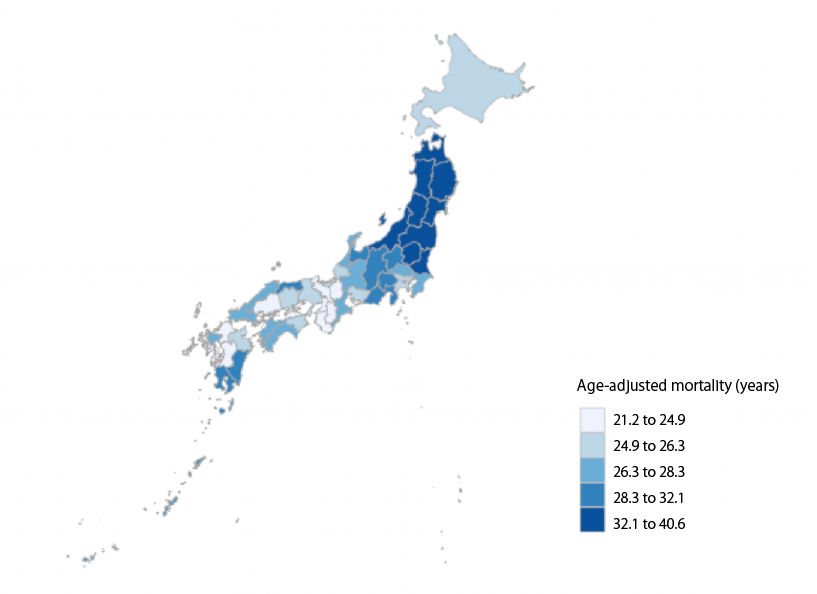
Average age-adjusted mortality from stroke between 2014 and 2019 in Japan by prefecture.

The overall mean age-adjusted mortality from stroke was 28.07 (SD 4.55) deaths per 100,000 for all prefectures in all data years. The average for each data year (in deaths per 100,000) was 30.62 (SD 4.49) in 2014, 29.40 (SD 4.41) in 2015, 28.28 (SD 4.04) in 2016, 27.68 (SD 4.22) in 2017, 26.60 (SD 4.28) in 2018, and 25.87 (SD 4.34) in 2019. The averages by prefecture are shown in Figure 1. The overall mean aging rate (expressed as a percentage) was 29.10 (SD 3.04) for all prefectures in all data years.

### Results of the Random Forest Analysis

**Figure 2:**
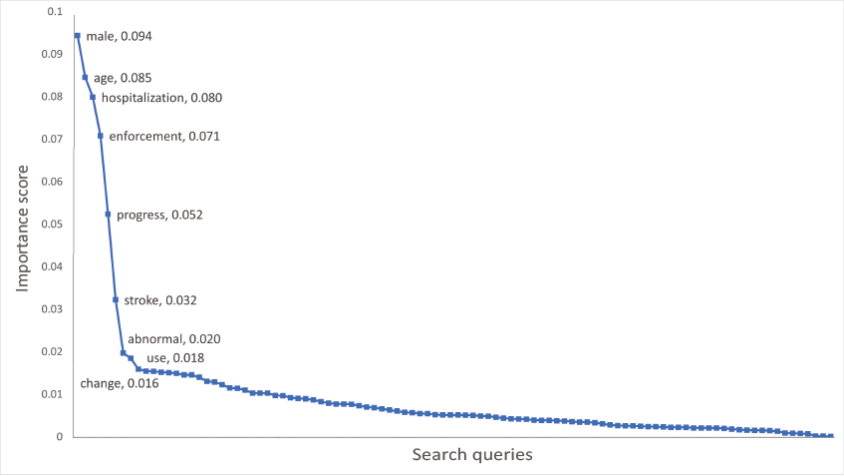
Importance scores of search queries in the random forest analysis with age-adjusted mortality from stroke as the outcome variable.

The accuracy score of the random forest regression was 89.94%, the average error was 2.79 degrees, and the mean squared error (MSE) value was 13.57 degrees. The best hyperparameters were as follows: n_estimators=40, bootstrap=True, criterion=MSE, max_depth=80, max_features=auto, min_samples_leaf=4, and max_samples_split=10. To identify the variables that contributed to the prediction, importance scores were calculated as shown in Figure 2. A total of 9 variables with a high importance score were selected as predictive variables for the regression analysis: male, age, hospitalization, enforcement, progress, stroke, abnormal, use, and change.

### Results of the Regression Analysis With the Generalized Linear Mixed Model

The Pearson correlation coefficients and VIF values for the variables used in the regression analysis are shown in Table 2. Of the absolute values of the correlation coefficient, the minimum and maximum values were 0.00 and 0.77, respectively, and the respective VIF values were 1.00 and 2.41. This suggested the absence of multicollinearity, and all variables were used in further analyses as predictors and control variables.

**Figure 3:**
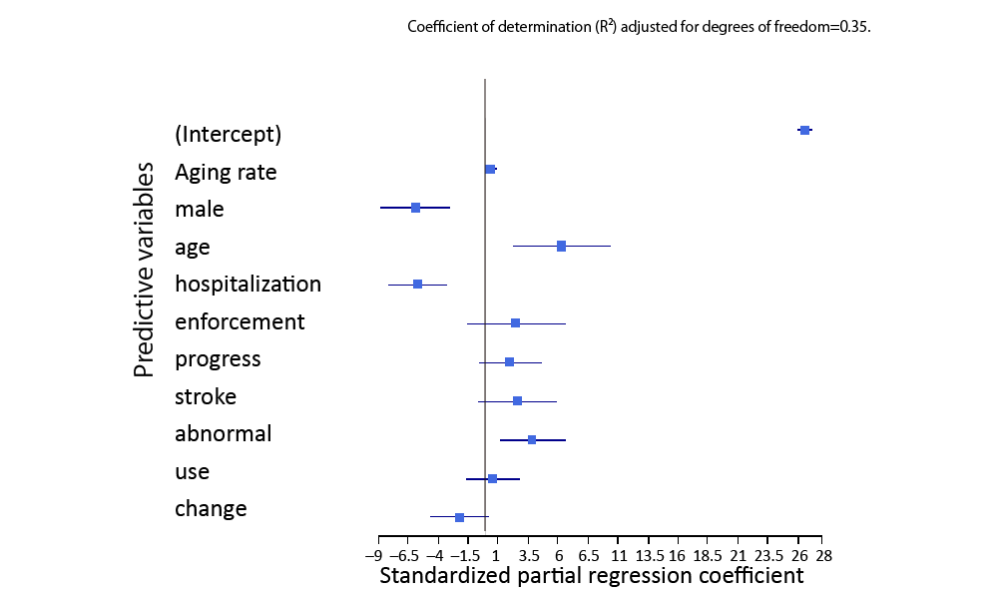
Results of the generalized linear mixed model with age-adjusted mortality from stroke as the outcome variable.

As a result of the regression analysis using the GLMM (Figure 3, Table 3), the standardized partial regression coefficients (β) and 95% confidence intervals showed that the following internet search engine queries were significantly associated with age-adjusted mortality from stroke: male (β=−5.83, 95% CI −8.67 to −3.29), age (β=−5.83, 95% CI −8.67 to −3.29), hospitalization (β=−5.83, 95% CI −8.67 to −3.29), and abnormal (β=3.83, 95% CI 1.14 to 6.56). Finally, the Rhat values, the number of effective samples representing the strength of autocorrelation, and Monte Carlo standard error suggested that the MCMC algorithm achieved convergence for all parameters.

**Table 2:** Correlation coefficients and variance inflation factors (VIFs) among variables in the generalized linear mixed model.

Variable		V1	V2	V3	V4	V5	V6	V7	V8	V9	V10	V11	V12	V13
**V1. Age-adjusted mortality**														
	*r*	1.00	−0.47	−0.35^**^	0.12	−0.26^**^	0.22^**^	−0.23^**^	0.27^**^	0.23^**^	0.07	0.10	0.11	−0.12
	P value	N/A^b^	<.001	<.001	.04	<.001	<.001	<.001	<.001	<.001	.25	.09	.06	.05
	VIF	N/A	1.29	1.14	1.02	1.08	1.05	1.06	1.08	1.05	1.00	1.01	1.01	1.01
**V2. Prefecture**														
	*r*	−0.47	1.00	0.00	0.06	−0.15^*^	−0.48^**^	−0.29^**^	−0.35^**^	−0.46^**^	−0.32^**^	−0.37^**^	−0.33^**^	−0.31^**^
	P value	<.001	N/A	>.99	.29	.01	<.001	<.001	<.001	<.001	<.001	<.001	<.001	<.001
	VIF	1.29	N/A	1.00	1.00	1.02	1.30	1.09	1.14	1.26	1.12	1.15	1.12	1.11
**V3. Data year**														
	*r*	−0.35^**^	0.00	1.00	0.35^**^	0.03	−0.04	0.11	−0.12^*^	−0.05	−0.12^*^	−0.05	−0.02	−0.05
	P value	<.001	>.99	N/A	<.001	.59	.50	.06	.04	.44	.04	.45	.77	.37
	VIF	1.14	1.00	N/A	1.14	1.00	1.00	1.01	1.01	1.00	1.02	1.00	1.00	1.00
**V4. Aging rate**														
	*r*	0.12	0.06	0.35^**^	1.00	−0.47^**^	−0.27^**^	−0.27^**^	−0.29^**^	−0.34^**^	−0.32^**^	−0.33^**^	−0.43^**^	−0.45^**^
	P value	.04	.29	<.001	N/A	<.001	<.001	<.001	<.001	<.001	<.001	<.001	<.001	<.001
	VIF	1.02	1.00	1.14	N/A	1.28	1.08	1.08	1.09	1.13	1.12	1.12	1.23	1.25
**V5. *Male*^a^**														
	*r*	−0.26^**^	−0.15^*^	0.03	−0.47^**^	1.00	0.52^**^	0.74^**^	0.43^**^	0.43^**^	0.66^**^	0.66^**^	0.51^**^	0.69^**^
	P value	<.001	.01	.59	<.001	N/A	<.001	<.001	<.001	<.001	<.001	<.001	<.001	<.001
	VIF	1.08	1.02	1.00	1.28	N/A	1.38	2.19	1.22	1.22	1.77	1.79	1.35	1.90
**V6. *Age***														
	*r*	0.22^**^	−0.48^**^	−0.04	−0.27^**^	0.52^**^	1.00	0.60^**^	0.76^**^	0.69^**^	0.71^**^	0.73^**^	0.63^**^	0.67^**^
	P value	<.001	<.001	.50	<.001	<.001	N/A	<.001	<.001	<.001	<.001	<.001	<.001	<.001
	VIF	1.05	1.30	1.00	1.08	1.38	N/A	1.56	2.37	1.89	2.02	2.11	1.67	1.79
**V7. *Hospitalization***														
	*r*	−0.23^**^	−0.29^**^	0.11	−0.27^**^	0.74^**^	0.60^**^	1.00	0.44^**^	0.42^**^	0.62^**^	0.65^**^	0.50^**^	0.66^**^
	P value	<.001	<.001	.06	<.001	<.001	<.001	N/A	<.001	<.001	<.001	<.001	<.001	<.001
	VIF	1.06	1.09	1.01	1.08	2.19	1.56	N/A	1.25	1.22	1.63	1.73	1.33	1.77
**V8. *Enforcement***														
	*r*	0.27^**^	−0.35^**^	−0.12^*^	−0.29^**^	0.43^**^	0.76^**^	0.44^**^	1.00	0.77^**^	0.64^**^	0.62^**^	0.67^**^	0.50^**^
	P value	<.001	<.001	.04	<.001	<.001	<.001	<.001	N/A	<.001	<.001	<.001	<.001	<.001
	VIF	1.08	1.14	1.01	1.09	1.22	2.37	1.25	N/A	2.41	1.70	1.64	1.81	1.34
**V9. *Progress***														
	*r*	0.23^**^	−0.46^**^	−0.05	−0.34^**^	0.43^**^	0.69^**^	0.42^**^	0.77^**^	1.00	0.53^**^	0.59^**^	0.66^**^	0.54^**^
	P value	<.001	<.001	.44	<.001	<.001	<.001	<.001	<.001	N/A	<.001	<.001	<.001	<.001
	VIF	1.05	1.26	1.00	1.13	1.22	1.89	1.22	2.41	N/A	1.40	1.54	1.76	1.42
**V10. *Stroke***														
	*r*	0.07	−0.32^**^	−0.12^*^	−0.32^**^	0.66^**^	0.71^**^	0.62^**^	0.64^**^	0.53^**^	1.00	0.66^**^	0.56^**^	0.59^**^
	P value	.25	<.001	.04	<.001	<.001	<.001	<.001	<.001	<.001	N/A	<.001	<.001	<.001
	VIF	1.00	1.12	1.02	1.12	1.77	2.02	1.63	1.70	1.40	N/A	1.78	1.46	1.53
**V11. *Abnormal***														
	*r*	0.10	−0.37^**^	−0.05	−0.33^**^	0.66^**^	0.73^**^	0.65^**^	0.62^**^	0.59^**^	0.66^**^	1.00	0.65^**^	0.61^**^
	P value	.09	<.001	.45	<.001	<.001	<.001	<.001	<.001	<.001	<.001	N/A	<.001	<.001
	VIF	1.01	1.15	1.00	1.12	1.79	2.11	1.73	1.64	1.54	1.78	N/A	1.72	1.60
**V12. *Use***														
	*r*	0.11	−0.33^**^	−0.02	−0.43^**^	0.51^**^	0.63^**^	0.50^**^	0.67^**^	0.66^**^	0.56^**^	0.65^**^	1.00	0.58^**^
	P value	.06	<.001	.77	<.001	<.001	<.001	<.001	<.001	<.001	<.001	<.001	N/A	<.001
	VIF	1.01	1.12	1.00	1.23	1.35	1.67	1.33	1.81	1.76	1.46	1.72	N/A	1.50
**V13. *Change***														
	*r*	−0.12	−0.31^**^	−0.05	−0.45^**^	0.69^**^	0.67^**^	0.66^**^	0.50^**^	0.54^**^	0.59^**^	0.61^**^	0.58^**^	1.00
	P value	.05	<.001	.37	<.001	<.001	<.001	<.001	<.001	<.001	<.001	<.001	<.001	N/A
	VIF	1.01	1.11	1.00	1.25	1.90	1.79	1.77	1.34	1.42	1.53	1.60	1.50	N/A

^a^The italicized variables are those with high importance scores in the random forest regression analysis with the Stroke 100 words.

^b^N/A: not applicable.

*P<.05, **P<.01.

**Table 3:** Standardized partial regression coefficient and 95% confidence intervals for the generalized linear mixed model with age-adjusted mortality from stroke as the outcome variable.

Predictive variable	Standardized partial regression coefficient (β)	95% CI
(Intercept)	26.6	26.08 to 27.20
Aging rate	0.34	0.18 to 0.87
Male	–5.83	–8.67 to –3.04
Age	6.31	2.32 to 10.34
Hospitalization	–5.69	–8.06 to –3.29
Enforcement	2.49	–1.52 to 6.54
Progress	1.99	–0.50 to 4.60
Stroke	2.63	–0.66 to 5.88
Abnormal	3.83	1.14 to 6.56
Use	0.55	–1.66 to 2.75
Change	–2.13	–4.55 to 0.21

## Discussion

### Principal Findings

The accuracy score of 89.94% in the random forest regression and the average error of 2.79 degrees indicate that a stroke-related query can predict age-adjusted mortality from stroke in each prefecture in Japan with high accuracy.

The regression analysis using the GLMM showed that the search terms “male” and “hospitalization” were associated with prefectures with low age-adjusted mortality from stroke, whereas the keywords “age” and “abnormal” were associated with prefectures with high age-adjusted mortality from stroke. Because the stroke risk increases with age and women tend to live longer, the absolute number of stroke events is higher in women [21]. However, the age-adjusted mortality rate is higher in men [22], and men tend to experience stroke at a younger age [23]. It is inferred that queries such as “male” and “age” became significant due to sex differences in stroke epidemiology. Moreover, because stroke outcome mainly depends on the time it takes to detect an abnormality early and to receive appropriate treatment, search terms such as “abnormal” or “hospitalization” may have been significant in our model. However, it is necessary to carefully examine the confounding factors of these significant queries, and it is desirable to analyze the mixed search of multiple queries and design a study that can demonstrate causality.

### Comparison With Prior Work

The results of the random forest analysis clarified that stroke was included in the query with a high importance score and that the query concerning the disease was associated with the age-adjusted death rate in Japan, confirming previous research in other countries [10-12]. In contrast to previous studies, this study was novel in that it extracted high-frequency words from abstracts related to stroke and analyzed the association between search behavior for a wide range of queries and age-adjusted mortality from stroke. As a result, queries such as “male”, “age”, and “hospitalization”, in addition to disease name and symptom, were confirmed to have a significant association with stroke-related mortality. Furthermore, in this study, we extracted high-frequency words from the abstracts of academic papers, but it may be possible to obtain more realistic results by analyzing high-frequency words extracted from the records of stroke patients.

### Limitations

This study has several limitations. Since this is an ecological study, it is possible to assess each region, but the impact on individuals living in the region cannot be determined. However, to address public health issues, it may be important to identify high-risk regions for stroke based on internet queries and alert the residents of those regions. Therefore, further studies on the criteria and timing of alerting based on information-seeking behavior are needed. In addition, considering the seasonal variation in age-adjusted mortality and the research number of stroke-related queries, the unit of analysis in this study was each prefecture in Japan with a large population. However, in order to propose specific administrative measures, it is desirable to conduct a more detailed analysis by region, such as by area classification based on the status of emergency transport and medical services.

### Conclusion

Stroke-related search behavior was associated with age-adjusted mortality from stroke in each prefecture in Japan. Query terms that were strongly associated with age-adjusted mortality rates of stroke suggest the possibility that individual characteristics such as sex and age have an impact on stroke-associated mortality and that it is important to receive medical care early after stroke onset. Further studies on the criteria and timing of alerting are needed by monitoring information-seeking behavior to identify queries that are strongly associated with stroke mortality.

## Appendix

Multimedia Appendix 1 Search queries related to the stroke signs and symptoms from the abstracts in Ichushi-Web.

## Abbreviations

GLMM: generalized linear mixed model
MAE: mean absolute error
MCMC: Markov chain Monte Carlo
MSE: mean squared error
Stroke 100: 100 frequently occurring words that were extracted from the abstract of Japanese academic papers related to stroke
VIF: variance inflation factor
