# Supplementary material for: Prediction of Age-Adjusted Mortality From Stroke in Japanese Prefectures: Ecological Study Using Search Engine Queries
Source: JMIR Form Res. 2022 Jan 20;6(1):e27805. doi: 10.2196/27805 (PMC8814924; doi:10.2196/27805)
Supplement: Multimedia Appendix 1 [file formative_v6i1e27805_app1.doc]

| No. | Query | Number of  occurrences |  | No. | Query | Number of  occurrences |
| --- | --- | --- | --- | --- | --- | --- |
| 1 | patient | 6841 |  | 51 | findings | 1368 |
| 2 | flock | 6449 |  | 52 | blood vessels | 1364 |
| 3 | case | 4903 |  | 53 | change | 1343 |
| 4 | stroke | 4659 |  | 54 | lesion | 1316 |
| 5 | walking | 4573 |  | 55 | difference | 1303 |
| 6 | paralysis | 4338 |  | 56 | pulse | 1296 |
| 7 | improvement | 4280 |  | 57 | report | 1272 |
| 8 | side | 3747 |  | 58 | acute | 1263 |
| 9 | cerebral infarction | 3590 |  | 59 | recovery | 1243 |
| 10 | hemiplegia | 3318 |  | 60 | independence | 1232 |
| 11 | onset | 3195 |  | 61 | intervention | 1225 |
| 12 | significant | 3136 |  | 62 | comparison | 1213 |
| 13 | symptoms | 3049 |  | 63 | postoperative | 1206 |
| 14 | treatment | 2937 |  | 64 | illness | 1206 |
| 15 | exercise | 2873 |  | 65 | age | 1199 |
| 16 | consideration | 2823 |  | 66 | suggestion | 1199 |
| 17 | hospitalization | 2762 |  | 67 | carotid artery | 1158 |
| 18 | function | 2745 |  | 68 | necessity | 1144 |
| 19 | target | 2644 |  | 69 | rate | 1079 |
| 20 | period | 2575 |  | 70 | item | 1072 |
| 21 | evaluation | 2505 |  | 71 | type | 1046 |
| 22 | author | 2391 |  | 72 | stimulus | 1036 |
| 23 | brain | 2317 |  | 73 | stenosis | 1036 |
| 24 | diagnosis | 2269 |  | 74 | clarity | 997 |
| 25 | motion | 2155 |  | 75 | relation | 996 |
| 26 | lower limbs | 2134 |  | 76 | status | 995 |
| 27 | disability | 2081 |  | 77 | bleeding | 978 |
| 28 | male | 2070 |  | 78 | abnormal | 975 |
| 29 | decline | 1958 |  | 79 | rank | 964 |
| 30 | average | 1881 |  | 80 | value | 954 |
| 31 | administration | 1865 |  | 81 | rehabilitation | 934 |
| 32 | technique | 1861 |  | 82 | week | 932 |
| 33 | start | 1761 |  | 83 | measurement | 925 |
| 34 | infarction | 1752 |  | 84 | disease | 919 |
| 35 | enforcement | 1703 |  | 85 | possibility | 909 |
| 36 | upper limbs | 1697 |  | 86 | blockage | 894 |
| 37 | implementation | 1690 |  | 87 | factor | 893 |
| 38 | training | 1676 |  | 88 | activities | 891 |
| 39 | female | 1618 |  | 89 | impact | 888 |
| 40 | artery | 1570 |  | 90 | state | 885 |
| 41 | test | 1566 |  | 91 | both side | 882 |
| 42 | appearance | 1553 |  | 92 | difficulty | 856 |
| 43 | possible | 1508 |  | 93 | muscle | 854 |
| 44 | discharge | 1489 |  | 94 | area | 848 |
| 45 | therapy | 1460 |  | 95 | blood clot | 847 |
| 46 | effect | 1417 |  | 96 | surgery | 846 |
| 47 | use | 1415 |  | 97 | refinement | 815 |
| 48 | progress | 1391 |  | 98 | clinical | 803 |
| 49 | ability | 1382 |  | 99 | disease | 797 |
| 50 | neck | 1374 |  | 100 | effectiveness | 788 |
